# Supplementary material for: Neuroscience Knowledge and Endorsement of Neuromyths among Educators: What Is the Scenario in Brazil?
Source: Brain Sci. 2022 Jun 2;12(6):734. doi: 10.3390/brainsci12060734 (PMC9221520; doi:10.3390/brainsci12060734)
Supplement: Supplementary file 1 [file brainsci-12-00734-s001.zip › brainsci-1674857-supplementary.pdf]

Supplementary Material

Table S1. Distribution of the survey

By email		
**Event**	**Region (state)**	**Number of people reached**
South region Symposium (Lecture)	SOUTH (RS)	68
Educators meeting in Manaus (Lecture)	NORTH (AM)	90
Educators meeting in Fortaleza (Lecture)	NORTHEAST (CE)	44
Pedagogical meeting in Recife (Lecture)	NORTHEAST (PE)	785
Pedagogical meeting in Rio de Janeiro (Lecture)	SOUTHEAST (RJ)	734
Pedagogical meeting in Londrina (Lecture)	SOUTH (PR)	240
Pedagogical meeting in São Paulo (Lecture)	SOUTHEAST (SP)	851
Educators meeting in Palmas (Lecture)	NORTH (TO)	101
25ª Municipal day for teaching and learning (Lecture)	SOUTH (RS)	102
Meeting of private school teachers	SOUTHEAST (SP)	48
Education meeting in Fortaleza (Lecture)	NORTHEAST (CE)	42
Education meeting in Recife (Lecture)	NORTHEAST (PE)	46
Education meeting in Porto Alegre (Lecture)	SOUTH (RS)	45
Education meeting in São Paulo (Lecture)	SOUTHEAST (SP)	118
**Via social media (FACEBOOK)**		
**Groups**	**Region (state)**	**Number of people reached**
Roraima professors' group	SOUTHEAST (SP)	3,000 followers
Pernambuco professors' group	NORTHEAST (PE)	28,800 followers
Tocantins professors' group	NORTH (TO)	698 followers
Alagoas state professors' group	NORTHEAST (AL)	4,000 followers
Elementary school professors' group	BRAZIL	288,700 followers
Professors syndicate group	NORTH & NORTHEAST	8,055 followers
Union of educational establishments in the state of São Paulo (Sieeesp)	SOUTHEAST (SP)	3,585 followers
**By Journals**		

Brazilian Association of Psychopedagogy	BRAZIL	
Federal Council of Education	BRAZIL	

Table S2 . Score by Region. N: number of participants in each group; Mean: mean scores for each group; SEM: standard error of the mean.

Region	N	Mean	SEM
South	219	86.62	0.43
Southeast	1155	87.36	0.19
Midwest	69	84.78	1.14
North	34	85.50	0.96
Northeast	157	85.55	0.55

Table S3 . Score by type of institution. N: number of participants in each group; Mean: mean scores for each group; SEM: standard error of the mean.

Type of institution	N	Mean	SEM
Municipal public school	520	86.42	0.30
State public school	176	86.46	0.50
Federal public school	36	88.79	1.39
Private school	582	87.40	0.26
NGO	44	85.39	1.11
Other	115	86.86	0.68
Multiple public schools	46	85.94	0.89
Multiple private schools	29	88.42	1.21
Multiple public + private schools	86	88.12	0.75

Table S4. Score for private vs. public institutions. N: number of participants in each group; Mean: mean scores for each group; SEM: standard error of the mean.

Type of institution	N	Mean	SEM
Public education	791	86.55	0.24
Private education	611	87.45	0.25
Both	232	86.93	0.47

Table S5. Score by teaching level. N: number of participants in each group; Mean: mean scores for each group; SEM: standard error of the mean.

Teaching level	N	Mean	SEM
Pre-school/kindergarten	307	85.66	0.39
First years of elementary school	417	86.65	0.33
Last years of elementary school	99	87.08	0.58
High school	67	87.15	0.98
Technical school	28	86.35	1.13
College/university	133	88.72	0.53

Multiple grades up to elementary	254	87.45	0.41
Multiple grades up to high school	212	86.75	0.47
Multiple grades up to technical school	14	87.24	1.28
Multiple grades up to college/university	103	88.59	0.66

Table S6. Score by time in education. Time in education: years; N: number of participants in each group; Mean: mean scores for each group; SEM: standard error of the mean.

Time on education	N	Mean	SEM
Less than a year	71	85.26	0.92
1-3	129	85.52	0.68
4-6	178	86.69	0.51
7-10	244	86.70	0.43
11-20	473	86.95	0.30
21-30	357	87.56	0.34
31-40	148	87.78	0.52
More than 40	34	88.55	0.96

Table S7. Score by age groups. Age: in years; N: number of participants in each group; Mean: mean scores for each group; SEM: standard error of the mean.

Age	N	Mean	SEM
21-30	172	86.73	0.57
31-40	492	86.60	0.29
41-50	549	87.14	0.28
51-60	331	87.13	0.38
61-70	79	86.88	0.72
71+	11	90.26	1.67

Table S8. Score by Gender. N: number of participants in each group; Mean (SEM): mean scores for each group (standard error of the mean); ND: Not declared.

Gender	N	Mean	SEM
Female	1454	86.92	0.17
LGBTTT	15	90.71	1.73
Male	162	87.08	0.53

Table S9. Score by institutional role. N: number of participants in each group; Mean: mean scores for each group; SEM: standard error of the mean.

Institutional role	N	Mean	SEM
Teacher	942	86.67	0.22
Coordinator	165	87.14	0.57
Principal	71	87.27	0.71
Advisor	53	87.80	0.76
Owner	2	91.07	5.36
Intern	39	87.27	0.95
Volunteer	34	88.44	1.09

Others	237	87.14	0.42
Multiple roles (administrative)	34	86.66	0.90
Multiple roles (teaching)	22	86.20	1.47
Multiple roles (admin + teaching)	35	88.67	1.18

Table S10. Confidence levels according to age. Age: in years; N: number of participants in each group; Mean: mean scores for each group; SEM: standard error of the mean.

Age	N	Mean	SEM
21-30	172	4.2	0.04
31-40	492	4.39	0.02
41-50	549	4.41	0.02
51-60	331	4.48	0.03
61-70	79	4.36	0.06
71+	11	4.35	0.16

Table S11. Confidence levels according to teaching level. N: number of participants in each group; Mean: mean scores for each group; SEM: standard error of the mean.

Teaching level	N	Mean	SEM
Pre-school/kindergarten	307	4.31	0.03
First years of elementary school	417	4.44	0.03
Last years of elementary school	99	4.33	0.05
High school	67	4.36	0.06
Technical school	28	4.23	0.10
College/university	133	4.41	0.04
Multiple grades up to elementary	254	4.47	0.03
Multiple grades up to high school	212	4.35	0.04
Multiple grades up to technical school	14	4.47	0.14
Multiple grades up to college/university	103	4.43	0.05

Table S12. Confidence levels according to time in education. Time in education: years; N: number of participants in each group; Mean: mean scores for each group; SEM: standard error of the mean.

Time on education	N	Mean	SEM
Less than a year	71	4.19	0.06
1-3	129	4.28	0.05
4-6	178	4.37	0.04
7-10	244	4.40	0.03
11-20	473	4.41	0.02
21-30	357	4.40	0.03
31-40	148	4.50	0.04
More than 40	34	4.42	0.09

Table S13. Score by individual questions. Differences among participants was tested using a two-way ANOVA. Significant p-values per variable were reported. Variables Age, gender, Type of institution, Institutional roles and Time in education did not present significant differences. Cap.: Capital; Pub.: Public; Priv.: Private.

Question	Region	Cap. vs outskirts	Pub. vs Priv.	Teaching levels	Category
We only use 10% of our brain's capacity (1)			p<0.045	p< 0.021	Brain characteristics
Learning maturation (cerebral maturity) depends exclusively on genetics (6)			p <0.003	p<0.038	Brain characteristics
Self-control training in students is exclusively the responsibility of parents, not teachers (7)		p<0.049			Executive and cognitive functions
Teachers should employ teaching methods that stimulate the right side (creative) or the left side (rational) depending on the type of student (8)				p<0.013	Learning strategies and methods
Left-handed students are not as capable of learning to read as right-handed students (11)	p<0.001				Literacy, reading and writing
Neuroimaging techniques like magnetic resonance imaging (MRI) can reveal a student's intelligence and capacity to learn (16)	p<0. 008				Learning strategies and methods
Dyslexia is a brain condition that is not related to intelligence; thus, an individual can be dyslexic as well as intelligent (17)	p<0.020			p<0.0007	Learning disorders
Male brains have greater capacity for logical reasoning (science, mathematics), while females are more intuitive (language, arts) (20)	p<0.014	p<0.002			Brain characteristics
The sleep cycle changes during adolescence; thus, pushing back the start time for morning classes could facilitate learning (21)	p<0.0003	p<0.020	p<0.024	p<0.0004	Neurophysiology and learning
Seven years old is considered a critical age for written language acquisition; thus, someone who does not learn to write by this age will most likely never become a good reader (24)		p<0.0324			Literacy, reading and writing
Learning to speak a second language during childhood (bilingualism) can compromise development (25)				p<0.0187	Literacy, reading and writing
Neuroplasticity (the brain's capacity to change, adapt and learn new things) ends after adolescence (27)	p<0.005				Brain characteristics
